# Influence of mode of delivery on neonatal QTc

**DOI:** 10.1038/s41372-026-02624-x

**Published:** 2026-03-16

**Authors:** Christina Eder, Stephan Gerling, Susanne Brandstetter, Markus-Johann Dechant, Angela Köninger, Annegret Schnabel, Christian Apfelbacher, Sven Wellmann, Michael Kabesch, the KUNO-Kids Study Group, Stephan Gerling, Sven Wellmann, Andreas Ambrosch, Petra A. Arndt, Andrea Baessler, Mark Berneburg, Romuald Brunner, Sara Fill Malfertheiner, André Franke, Robert Häsler, Iris Heid, Stefanie Heinze, Wolfgang Högler, Sebastian Kerzel, Michael Koller, Michael Leitzmann, Áine Lennon, David Rothfuß, Martin Promm, Bianca Schaub, Stephan Weidinger, Holger Michel

**Affiliations:** 1 University Children’s Hospital Regensburg (KUNO), University of Regensburg, Hospital St. Hedwig of the Order of St. John, Regensburg, Germany; 2 Clinic of Obstetrics and Gynecology St. Hedwig, University of Regensburg, Regensburg, Germany; 3 Institute of Social Medicine and Health Economics, University of Magdeburg, Magdeburg, Germany; 4 Institute of Laboratory Medicine, Microbiology and Hygiene, Barmherzige Brüder Hospital, Regensburg, Germany; 5 ZNL Transfercenter of Neuroscience and Learning, University of Ulm, Ulm, Germany; 6 Department of Internal Medicine II, Regensburg University Medical Center, Regensburg, Germany; 7 Department of Dermatology, University Medical Centre Regensburg, Regensburg, Germany; 8 Clinic of Child and Adolescent Psychiatry, Psychosomatics and Psychotherapy, Bezirksklinikum Regensburg (medbo), Regensburg, Germany; 9 Institute of Clinical Molecular Biology, Christian-Albrechts-University of Kiel, Kiel, Germany; 10 Department of Dermatology and Allergy, Christian-Albrechts-University of Kiel, Kiel, Germany; 11 Department of Genetic Epidemiology, University of Regensburg, Regensburg, Germany; 12 Bavarian Health and Food Safety Authority (LGL) Munich, Munich, Germany; 13 Department of Pediatrics and Adolescent Medicine, Johannes Kepler University Linz, Linz, Austria; 14 Center for Clinical Studies, University Hospital Regensburg, Regensburg, Germany; 15 Department of Epidemiology and Preventive Medicine, University of Regensburg, Regensburg, Germany; 16 Department of Conservative Dentistry and Periodontology, University Hospital Regensburg, University of Regensburg, Regensburg, Germany; 17 City of Regensburg, Coordinating Center for Early Interventions, Regensburg, Germany; 18 Department of Pediatric Urology, University Medical Center, Regensburg, Germany; 19 Pediatric Allergology, Department of Pediatrics, Dr. von Hauner Children’s Hospital, University Hospital, LMU Munich, Munich, Germany; 20 Department of Dermatology, Venereology and Allergy, University Hospital Schleswig-Holstein, Campus Kiel, Kiel, Germany

**Keywords:** Medical research, Risk factors

## Abstract

**Objective:**

To evaluate the influence of mode of delivery on repolarisation and QT interval in the neonatal electrocardiogram (ECG).

**Study design:**

KUNO-Kids is a prospective, population-based birth cohort study. Neonates received an ECG within the first week of life; subgroups were divided according to mode of delivery. Effects on neonatal QT interval were tested by linear and logistic regression analyses, adjusting for age at ECG recording.

**Results:**

In total, 712 neonates were included. The primary caesarean section group showed a significant higher mean QTc (regression coefficient 6.03, *p* = 0.033) and a significantly increased rate of prolonged QTc >450 ms (odds ratio 2.4, *p* = 0.042), compared to the spontaneous delivery group.

**Conclusion:**

The mode of delivery has a significant effect on neonatal QTc. Clinicians should consider this influence when deciding on postnatal and follow-up examinations, regarding additional risk factors for acquired long QT interval.

## Introduction

A severely prolonged QT interval is associated with life-threatening complications such as stillbirth and sudden infant death syndrome (SIDS) in infants as well as harmful cardiac arrhythmias and sudden unexplained death (SUD) in children and adults [1–5]. About 10% of SIDS cases can be attributed to long QT syndrome (LQTS) [3], a genetic cardiac ion channel disorder with a prevalence of approximately 1:2000 [6], for which mutations in 17 genes have been described [7]. In addition to genetically determined LQTS, acquired LQTS has been described. QT prolongation based on various external influences and pathophysiological mechanisms increase the risk of critical cardiac arrhythmias [8].

In neonates, studies have described transient postnatal QT interval prolongation with normalisation in most cases over the first days of life [9, 10]. Mechanisms of these prolongations and the resulting temporary proarrhythmogenic risk remain unclear. While drug induced QT prolongation is a major contributor to acquired LQTS in older patients [8], previous studies in the KUNO-Kids health cohort showed no association of maternal QT-prolonging medication and neonatal QT prolongation [11]. Based on previous exploratory analysis, we hypothesized that delivery mode may influence the QT interval. Although the influence of several maternal and perinatal factors on the neonatal ECG has already been investigated, data on the effect of delivery mode on the QT interval in neonates is scarce and inconclusive [12, 13]. Thus, the aim of this study was to further analyse the possible effect of mode of delivery on repolarisation and QT interval in the neonatal electrocardiogram (ECG) after birth in a large comprehensive birth cohort.

## Material and methods

### Study design and study population

The KUNO-Kids health study is a population-based prospective birth cohort study conducted at the Clinic St. Hedwig, Regensburg, Germany, as previously described [14]. Written informed consent was obtained for each case. The study was approved by the Ethics Committee of the University of Regensburg (14-101-0347; 19-1646-101).

Eligible for this analysis were neonates born between 1st of January 2018 until 31st of December 2019. We included all neonates with a postnatal ECG in the first week of life and complete documentation of delivery mode, maternal and neonatal demographics. Mothers with an outpatient birth, stillbirth, maternal age under 18 or maternal knowledge of German that was not sufficient for informed consent were excluded.

### Delivery mode and perinatal demographics

The mode of delivery was determined based on the documentation in the patient file and differentiated into uncomplicated vaginal delivery, primary caesarean section (without previous labour), secondary caesarean section (with previous labour) and vaginal operative delivery (vacuum extraction, forceps delivery).

Data on the demographic characteristics of the mother (age, height, weight, weight gain during pregnancy), on the characteristics of the newborn (gestational age, birth weight, birth length, sex) and on perinatal characteristics (pH and base excess on the umbilical cord, APGAR score after 1, 5 and 10 min) as well as on the age of the infant at the time of the ECG were collected on the basis of patient records and a postnatal interview with the mother. The data are given in Table 1.

**Table 1:** Baseline characteristics grouped by mode of delivery (n = 712 participants).

	Total *n* = 712	Sponta-neous delivery *n* = 399	Primary caesarean section *n* = 99	Secondary caesarean section *n* = 148	Vaginal operative delivery *n* = 66	Signifi-cance (*p*-value)
Maternal age, years	32.4 (4.4)	32.1 (4.3)	33.2 (4.7)	33.4 (4.5)	31.1 (3.8)	<0.001
Maternal height, cm	167 (6.1)	168 (5.8)	167 (6.8)	167 (6.1)	167 (7.0)	0.073
Maternal weight, kg	68 (14.8)	68 (14.8)	70.3 (17.2)	67.7 (13.6)	65 (13.8)	0.129
Gestational age, weeks	39.5 (1.6)	39.7 (1.4)	38.4 (1.4)	39.6 (1.8)	40 (1.6)	<0.001
Birth weight, g	3358 (505)	3410 (464)	3343 (578)	3348 (572)	3382 (465)	0.495
Birth height, cm	51.5 (2.5)	51.6 (2.4)	51.0 (2.9)	51.4 (2.8)	51.7 (2.5)	0.219
Sex of the infant female / male *	349 / 363	203 / 196	46 / 53	68 / 80	32 /34	0.712
Umbilical cord arterial pH	7.25 (0.08)	7.24 (0.08)	7.31 (0.04)	7.28 (0.1)	7.19 (0.07)	<0,001
Umbilical cord arterial base excess	−4.93 (3.0)	−5.59 (2.8)	−2.40 (1.8)	−3.89 (2.6)	−6.99 (3.2)	<0,001
APGAR 1 minute	8.8 (0.9)	8.9 (0.6)	8.9 (1.0)	8.4 (1.3)	8.4 (1.0)	<0,001
APGAR 5 minutes	9.7 (0.7)	9.8 (0.5)	9.7 (0.7)	9.4 (0.9)	9.5 (0.9)	<0,001
APGAR 10 minutes	9.9 (0.4)	9.9 (0.2)	9.9 (0.4)	9.8 (0.5)	9.9 (0.4)	<0,001

Continuous variables expressed as mean ± SD (except * which are total numbers).

### ECG

In total, 12 lead ECGs were recorded in the first week of life following standard operating procedures as described before [10] at a paper speed of 50 mm/s including an additional rhythm recording at 25 mm/s. We used a standard device (MAC 5500 HD®, GE Healthcare, Freiburg, Germany) and ten adhesive electrodes (Ambu® BlueSensor NF50-A/12, Ambu, Bad Nauheim, Germany). All ECG recordings were analysed by experienced paediatric cardiologists (SG, HM), who were blinded to the mode of delivery.

In the presented study, we analyzed heart rate, rhythm, frontal QRS axis, QRS duration, frontal T axis and pattern of precordial leads with T wave inversion and length of QT interval with respect to delivery mode. As recommended in the guidelines for the interpretation of the newborn ECG of the European Society of Cardiology [15] the QT interval was measured manually from the beginning of the Q wave to the end of the T wave using the tangent method. For time correction (QTc) we used Bazett’s formula (QTc [ms] = QT interval [ms] / (√RR [s] / 1[s])). The QTc value was measured in lead II, and if the value was > 440 ms, a mean of lead II, V5 and V6 was calculated according to Schwartz et al. [15].

### Data management

Parental and neonatal demographics, perinatal characteristics and ECG parameters were entered in an electronic case report form (eCRF) and extensive plausibility checks were performed. The collected data was stored pseudonymized on a protected study server [14]. As mentioned above, further patient information regarding mode of delivery and further medical treatment was determined using the SAP hospital software.

### Statistical analysis

We used IBM SPSS Statistics (Version 28) for descriptive statistic, univariate and multivariate regression analysis and Pearson’s chi-squared test. As suggested by Schwartz et al. [6] we used 450 ms as the cut-off value in logistic regression analysis to test whether delivery mode was associated with a prolonged QTc-interval.

## Results

A total of 712 neonates were included in the analysis. There was an approximately equal sex distribution with 349 (49%) girls. The participants were born after a mean duration of pregnancy of 39.5 weeks (SD 1.6) with a mean weight of 3358 g (SD 505) and a mean height of 51.5 cm (SD 2.5). The rate of spontaneous delivery was 56% (*n* = 399), primary caesarean section was performed in 13.9% (*n* = 99), secondary caesarean section in 20.8% (*n* = 148) and vaginal operative delivery in 9.3% (*n* = 66). Demographic characteristics of the overall study group and the subgroups are given in Table 1.

ECG findings for the overall cohort and subgroups are shown in Table 2. The mean age at ECG recording was 1.8 days (SD 0.92), with lower values in spontaneous delivery group and higher values in both caesarean section groups. As those values differed significantly in the primary and secondary caesarean section group compared to the spontaneous delivery group (*p* < 0.001), we adjusted the multivariate analyses for all ECG parameters for the age of the neonates. For the overall cohort, the mean heart rate was 119 bpm (SD 17), the mean QRS axis was 131° (SD 35), the mean QRS duration was 59 ms (SD 3.0) and the mean T axis was 59° (SD 26). There was no significant association between the mode of delivery and any of the ECG parameters.

**Table 2:** ECG findings grouped by mode of delivery (*n* = 712 participants).

	Total *n* = 712	Spontaneous delivery *n* = 399	Primary caesarean section *n* = 99	Secondary caesarean section *n* = 148	Vaginal operative delivery *n* = 66
Mean age at the ECG in days	1.8 (0.92)	1.64 (0.81)	2.05 (1.05) *<0.001*	2.13 (1.04) *<0.001*	1.8 (0.77) *0.162*
Mean heart rate, beats per minute	119 (17)	118 (17)	122 (15) *0.066*	119 (17) *0.756*	120 (19) *0.344*
Mean QRS axis, degrees	131 (35)	133 (35)	127 (37) *0.141*	127 (30) *0.077*	138 (37) *0.293*
Mean QRS duration, ms	59.0 (3.0)	59.3 (3.2)	59.2 (3.5) *0.816*	59.6 (4.0) *0.444*	59.9 (3.1) *0.150*
Mean T axis, degrees	59 (26)	62 (24)	57 (30) *0.425*	54 (27) *0.054*	60 (27) *0.689*
Mean QTc interval, ms	416 (25)	415 (24)	420 (25) *0.033*	416 (24) *0.409*	417 (28) *0.458*
QTc > 450 ms *	5.3% (38)	4.3% (17)	9.1% (9) *0.042*	4.7% (7) *0.674*	7.6% (5) *0.223*

Continuous variables expressed as mean ± SD (except * which are percentages and total numbers).

*P* values for comparisons with the spontaneous delivery group are represented in *italics*.

*ECG* electrocardiogram, QTc corrected QT interval using Bazett’s formula.

**Fig. 1:**
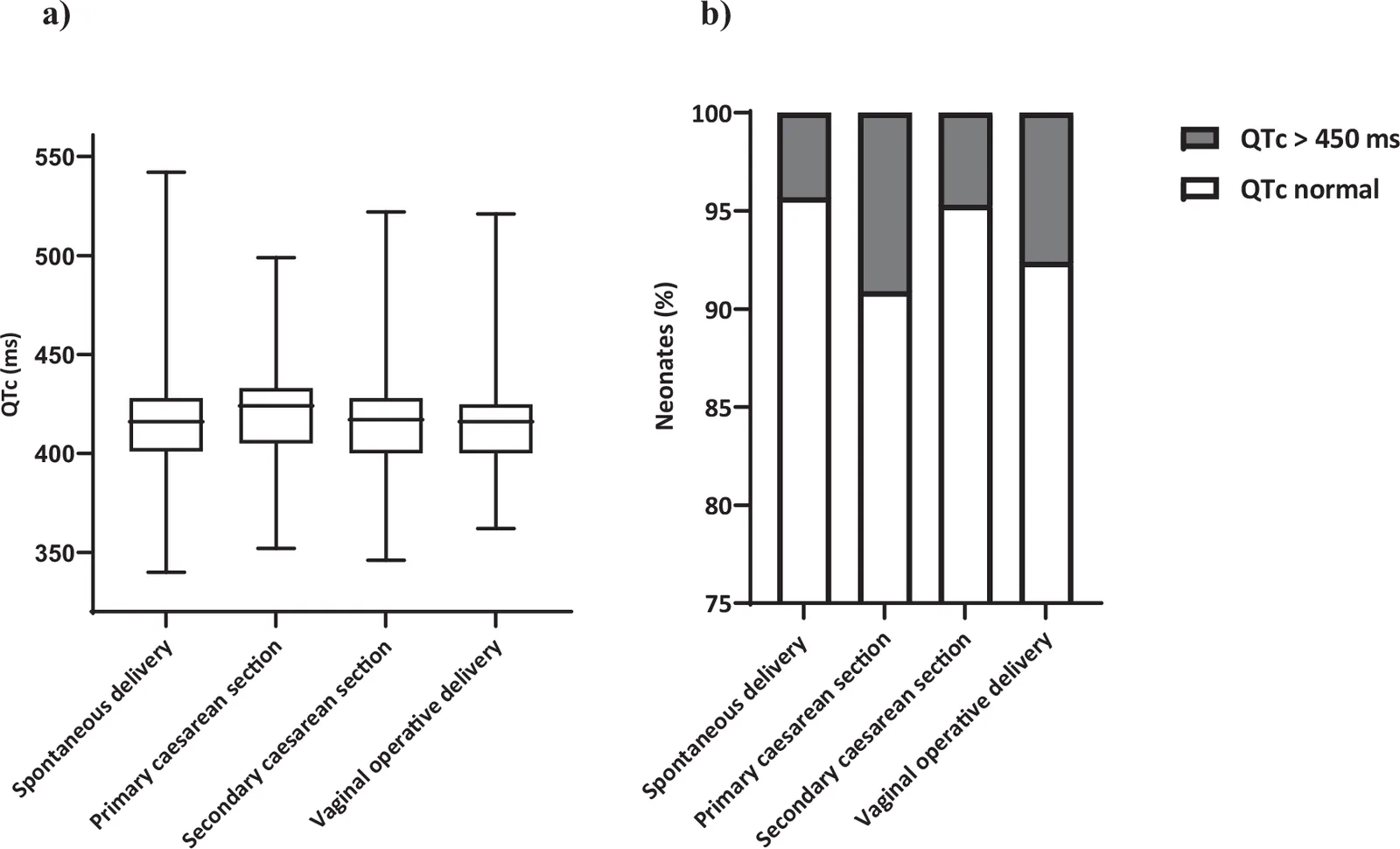
Increased mean QTc interval and rate of prolonged QTc >450 ms in primary caesarean section. **a** Boxplot of the neonatal QTc intervals of neonates grouped by mode of delivery. **b** Bar chart illustrating the percentage distribution and ratio of normal QTc interval to QTc >450 ms grouped by mode of delivery: spontaneous delivery 4.3% (*n* = 17), primary caesarean section 9.1% (*n* = 9), secondary caesarean 4.7% (*n* = 7) and vaginal operative delivery 7.6% (*n* = 5).

Other results were found for the QTc. The mean QTc of the overall cohort was 416 ms (SD 25 ms). In the subgroups the mean QTc was 415 ms (SD 24) for the spontaneous delivery group, 420 ms (SD 25) for the primary caesarean section group, 416 ms (SD 24) for the secondary caesarean section group and 417 ms (SD 28) for vaginal operative delivery group. After adjustment for the age of the neonate in a multivariate regression analysis mean QTc was significantly higher in the primary caesarean section group, compared to the spontaneous delivery group (coefficient 6.03, CI 0.5–11.6, *p* = 0.033). The secondary section group and vaginal operative delivery group did not show any significant difference compared to the spontaneous delivery group. The data are shown in Table 3 and Fig. 1.

**Table 3:** Multivariate linear regression analysis on the effect of mode of delivery on the neonatal QT interval, correcting for the age of the neonate at the ECG recording.

	Unstandardized coefficient		Standardized coefficient		Significance (*p*value)
	B 95% (confidence interval)	Std. error	Beta	t	
Constant	418 (414–422)	2.09		200	<0.001
Primary caesarean section	**6.03 (0.50–11.55)**	**2.81**	**0.085**	**2.14**	**0.033**
Secondary caesarean section	2.00 (−2.76–6.76)	2.42	0.033	0.83	0.409
Vaginal operative delivery	2.42 (−4.00 to 8.83)	3.26	0.029	0.74	0.458
Age at ECG (days)	−1.98 (−4.01 to 0.05)	1.03	−0.074	−1.91	0.056

The spontaneous delivery group serves as the reference group.

Statistically significant data points are represented in **bold** type.

The QTc was >450 ms in 38 neonates (5.3%), overall. In the subgroups a prolonged QTc was found in 4.3% of the spontaneous delivery group, 9.1% of the primary caesarean section group, 4.7% of the secondary caesarean section group and 7.6% of the vaginal operative delivery group (Fig. 1). Logistic regression analysis showed a significantly increased rate of prolonged QTc for the primary caesarean section group compared to spontaneous delivery (odds ratio 2.4, *p* = 0.042). The other group comparisons showed no significant difference (Table 4).

**Table 4:** Logistic regression analysis on the effect of mode of delivery on QT interval prolongation (QTc > 450 ms), correcting for the age of the neonate at the ECG recording.

	Odds ratio	95% confidence interval		Significance (*p*value)
		Lower bound	Upper bound	
Constant	0.060			<0.001
Primary caesarean section	**2.421**	**1.031**	**5.681**	**0.042**
Secondary caesarean section	1.217	0.487	3.046	0.674
Vaginal operative delivery	1.905	0.676	5.369	0.223
Age at ECG (days)	0.826	0.559	1.222	0.339

The spontaneous delivery group serves as the reference group.

Statistically significant data points are represented in **bold** type.

## Discussion

In the present prospective population-based study we analysed ECGs from 712 neonates during the first seven days of life. As a new finding of this study, we were able to demonstrate an association between the mode of delivery and QT interval duration. The mean QTc was significantly higher in the primary caesarean section group than in the spontaneous delivery group and a significantly higher rate of prolonged QTc was observed in the primary caesarean section group. Given the substantial variability of the QT interval during the first week of life, we adjusted our analyses accordingly. Even after taking the age at ECG recording into account, the results remained consistent.

Overall, both our descriptive statistics and ECG findings were consistent with previously published data and baseline characteristics of our study participants were comparable to those of other large studies that examined maternal and perinatal factors influencing the neonatal ECG [12]. Possibly reflecting obstetric decisions based on maternal and perinatal factors, there was a significant difference in maternal age and gestational age in the subgroups of our study. The question arises whether these differences might have an influence on the respective QTc values. The Copenhagen Baby Heart study showed no correlation between QTc and maternal age in a very large cohort [16]. Data on influence of gestational age on QTc is inconclusive. While Hartmann et al. showed a modest effect of gestational age on QTc with no significant association after multivariate adjustment in the Danish cohort [17], Séguéla et al. reported an effect of gestational age on the QTc values that was pronounced in preterm infants around 32 weeks of gestation, which is outside the range of our study cohort (33–43 weeks) [18]. An Italian study again found no significant difference in QTc length depending on gestational age, including term and preterm infants [13]. Hence, data on the effect of gestational age on QTc values from previous studies are inconclusive. Despite our large overall study cohort, the number of children in individual subgroups did not allow correction for multiple confounders, so we limited the adjustment to the age of the children at the timing of the ECG recording as a known important factor influencing the QT interval. Therefore, we cannot rule out the possibility that the slightly lower gestational age in the primary section group compared to the spontaneous delivery group in our study cohort (38.4 vs 39.7 weeks of GA) might have a confounding effect on the QTc values. Postnatal neonatal adaption values (APGAR, base excess and pH) were also different in the subgroups of our cohort with all values within the normal range [19, 20]. The slightly higher pH and lower base excess observed in the primary caesarean section group may reflect the absence of pre-labour contractions and altered postnatal adaption process.

Heart rate, QRS axis, and T wave inversion were within the normal reference ranges as outlined in the European Society of Cardiology guidelines for the interpretation of neonatal electrocardiograms [15]. The values on the T axis correspond to those published in the American Journal of Cardiology. This publication examines the evolution and significance of T wave changes in normal newborns during the first seven days of life [21]. No significant association could be demonstrated for delivery mode and any of these ECG parameters. This indicates that QT prolongation occurs as an isolated finding rather than in conjunction with other ECG alterations such as QRS prolongation or change in T wave morphology.

To our knowledge, the existing literature provides no pathophysiological concept to explain the reported influence of mode of delivery on neonatal QTc. One possible explanation seems to be that the significantly longer QTc in the group with primary caesarean section is the result of an altered circulatory transition process. Postnatal, the circulatory system transitions from foetal to neonatal. This leads to numerous changes in the cardiovascular and respiratory systems. Altered foetal stress and the associated neurohumoral activation depending on the mode of delivery can influence this transition [11]. In this study, we found delivery-mode related differences in postnatal transition characteristics, as previously mentioned (APGAR scores and umbilical cord pH). Since neonates with clinically relevant respiratory distress syndrome were excluded from this study, these findings could reflect clinically subtle differences in the postnatal adaptation. Previous studies showed that circulatory adaptation of neonates delivered vaginally is completed earlier [22]. An increased risk for a prolonged or impaired postnatal transition has been described in particular for infants born by elective caesarean section without prior onset of labour [23–25]. In addition, it has been demonstrated that a cumulative effect of several other maternal and perinatal risk factors for neonatal transition can lead to a prolonged neonatal QT interval [12]. It is conceivable that pathophysiological processes associated with an altered postnatal transition in children born by primary caesarean section may influence the QT interval, although the underlying mechanism remains unclear.

Additionally, different maternal medications are administered during caesarean section compared to spontaneous delivery. Medications are a common cause of acquired long QT syndrome, which is characterised by prolonged QT intervals caused by environmental factors [26]. A previous publication of our cohort study investigated the influence of maternal medication on the QTc. No statistically significant effect was found. However, the study did not specifically examine drugs administered at birth. Instead potential QT interval-prolonging drugs taken by the mother in the last four weeks of pregnancy, known for placental transfer, were examined [11]. By removing the stressor, i.e. by delivery of the neonate and correspondingly elimination of potentially influencing medication, the QTc time would be expected to normalise.

It remains to be discussed whether the increased QT interval and the higher rate of prolonged QTc in the primary caesarean section group are clinically relevant. It has already been described, that transient postnatal QT prolongation with marked variability during the first days of life are followed by subsequent normalization [9, 10]. Schwartz et al. point out that the QT interval normalises in 40–50% of newborns within the first year of life and that more than 90% are genotype-negative for long QT syndrome after one year [27]. Similarly, in our study cohort, the initially prolonged QT intervals normalised in the follow-up examinations, which were conducted as described by Simma et al. [10]. Accordingly, no child with long QT syndrome was identified in this study cohort, suggesting that the observed QT prolongation was acquired and transient, occurring in the context of multiple risk factors such as perinatal stress, genetic susceptibility and possibly delivery mode related.

Acquired long QT syndrome has been shown to be related with critical cardiac arrhythmia [8] and the first postnatal days of life have been identified as a vulnerable time period for sudden unexpected early neonatal death [28]. Therefore, Pærregaard et al. recommended in a recent study, that QT interval abnormalities detected in neonates younger than one week should be reassessed after the first week of life [29]. In addition to other maternal and perinatal risk factors [12] and possible genetic susceptibility to acquired QT interval prolongation [26] based on the data in the presented study the mode of delivery should be taken into account in postnatal risk assessment. For neonates delivered by primary caesarean section who have additional risk factors for acquired long QT syndrome, postnatal ECG recording and depending on the results further monitoring should be considered. This allows children with acquired prolonged QT interval or possible long QT syndrome to be identified, ensuring that early treatment is not prevented and reducing the potential risk of ventricular arrhythmias and SIDS.

The strengths of the current study are the large study population, the standardised evaluation of the 12-lead ECGs and the uniform collection and amount of maternal and perinatal data. However, there are limitations to our study. Despite the considerably large cohort, the number of infants in the respective subgroups was considerably small. Ideally, all ECGs should be recorded at the same time interval after completion of essential adaptation processes of the neonatal circulation. Since this was not easily achievable in everyday clinical practice, a defined age of 0–7 days after birth was accepted. The age was then adjusted in the multivariate regression analysis. Further studies with repetitive ECG examinations are needed to describe the short-term dynamics of postnatal QT prolongation, particularly in relation to the mode of delivery and other perinatal risk factors, to enable a more accurate assessment of the potential risk of arrhythmia. Furthermore, our results might be biased because of the single-centre study design as delivery mode distribution, local socioeconomic profile of mothers, and perinatal characteristics may not be transferable to the general population.

## Conclusion

This study is the first to demonstrate the influence of the delivery mode on neonatal QTc. In our cohort, the QTc interval as well as the rate of prolonged QTc of > 450 ms were significantly higher in the primary caesarean section group in comparison to the spontaneous delivery group. The effect of the delivery mode remained significant after controlling for the neonatal age at ECG recording. While obstetric decisions regarding the mode of delivery follow professional requirements and guidelines, the results of this study, together with those of previous studies [22–25], indicate a possible negative impact on postnatal adaption following delivery by elective caesarean section. They underscore the importance of postnatal ECG recording, especially if there are additional risk factors for acquired QT interval prolongations. Even though gestational age does not appear to significantly influence the neonatal ECG, preterm infants delivered by primary caesarean section may still warrant particular attention. In addition to the mode of delivery, there are other pre-, peri- and postnatal factors that might have an influence on the neonatal QT interval. More data are needed to consider them appropriately in future studies or when interpreting neonatal ECGs in a clinical setting.

## Data Availability

The raw data supporting the conclusions of this article will be made available by the authors, without undue reservation.
